# Ecology and geography of avian influenza (HPAI H5N1) transmission in the Middle East and northeastern Africa

**DOI:** 10.1186/1476-072X-8-47

**Published:** 2009-07-20

**Authors:** Richard AJ Williams, A Townsend Peterson

**Affiliations:** 1 Natural History Museum and Biodiversity Research Center, The University of Kansas, Lawrence, Kansas 66045, USA

## Abstract

**Background:**

The emerging highly pathogenic avian influenza strain H5N1 ("HPAI-H5N1") has spread broadly in the past decade, and is now the focus of considerable concern. We tested the hypothesis that spatial distributions of HPAI-H5N1 cases are related consistently and predictably to coarse-scale environmental features in the Middle East and northeastern Africa.

We used ecological niche models to relate virus occurrences to 8 km resolution digital data layers summarizing parameters of monthly surface reflectance and landform. Predictive challenges included a variety of spatial stratification schemes in which models were challenged to predict case distributions in broadly unsampled areas.

**Results:**

In almost all tests, HPAI-H5N1 cases were indeed occurring under predictable sets of environmental conditions, generally predicted absent from areas with low NDVI values and minimal seasonal variation, and present in areas with a broad range of and appreciable seasonal variation in NDVI values. Although we documented significant predictive ability of our models, even between our study region and West Africa, case occurrences in the Arabian Peninsula appear to follow a distinct environmental regime.

**Conclusion:**

Overall, we documented a variable environmental "fingerprint" for areas suitable for HPAI-H5N1 transmission.

## Background

Highly pathogenic avian influenza of the strain H5N1 (hereafter "HPAI-H5N1") has received considerable attention as an emerging virus with human pandemic potential [1,2] since it was first shown to be the cause of human morbidity and mortality in Hong Kong in 1997 [3]. To date, however, its most serious impacts have been on domestic poultry: millions of domestic birds have been killed by HPAI-H5N1 infection, and >230 million domestic birds have been culled to contain the spread of the virus [4]. In contrast to the dramatic publicity, relatively few human cases are confirmed: at the time of writing, 385 human HPAI-H5N1 cases had been documented, of which 243 (63.1%) were fatal [5], from 60 countries [6]. Human cases however, may eventually prove to be significantly underreported, reducing case-fatality rates.

Until Spring 2005, HPAI-H5N1 was restricted to East and Southeast Asia [6]. Between May and June 2005, however, >6000 birds of 8 wild waterbird species were found dead at Qinghai Lake, in central China: HPAI-H5N1 was detected in 15 birds of 6 wild species [7], some migratory, fueling fears of broader spread [8]. This event apparently marked a turning point in the spread of the virus: by early 2006, it had been detected widely across South Asia, Western Europe, and parts of Africa [6]. However, whether this rapid spread resulted from accelerated dispersal or from improved surveillance detecting existing infections remains debatable [9].

The first Middle Eastern detection of HPAI-H5N1 was in Turkey in October 2005, in a flock of "backyard" turkeys (see ional file 1). Further detections followed in 7 Balkan countries (Bosnia-Herzegovina, Bulgaria, Croatia, Greece, Romania, Serbia and Montenegro, and Slovenia; November 2005 – March 2006), more broadly in the Middle East (Egypt, Iraq, Iran, Israel, Jordan, Kuwait, Palestinian Territories; November 2005 – March 2006), and the Caucasus (Azerbaijan and Georgia; January – February 2006) by March 2006. The virus was detected in Sudan and Djibouti in April 2006, and in Saudi Arabia in March 2007 [6]. Countries in the region yet to record cases include the richest (Bahrain, Qatar, United Arab Emirates) and the poorest (Eritrea, Somalia, Yemen).

**Figure 1:**
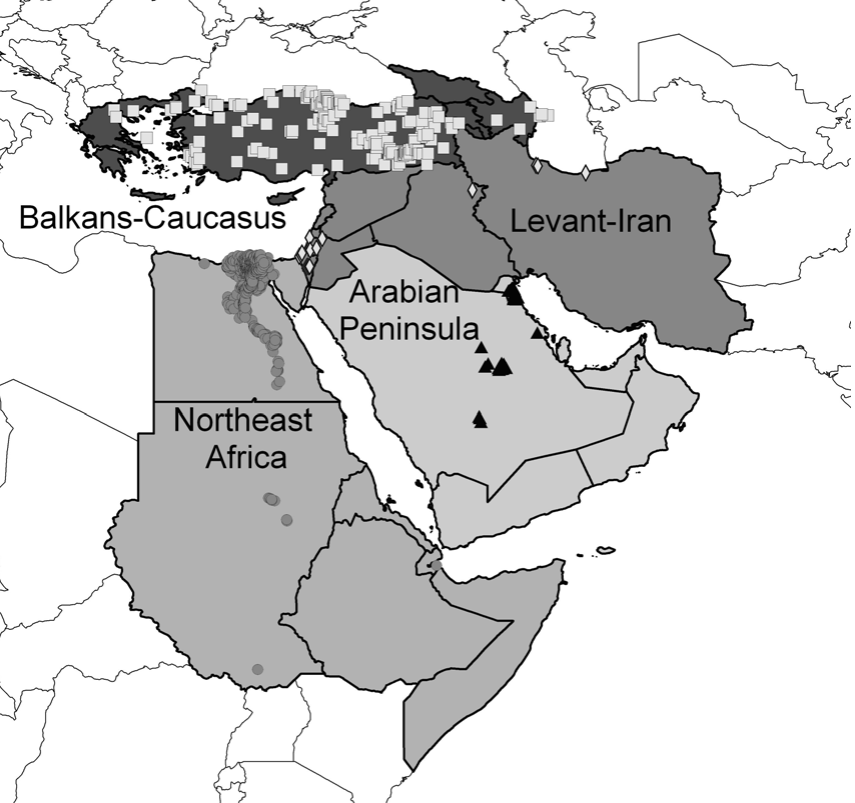
**Occurrence data for HP-H5N1 in the Middle East and northeastern Africa, and regional divisions used in this study**.

The concept of ecological niche describes the distinct ecological requirements that determine occurrences of organisms and other biological phenomena (including disease transmission, such as HPAI-H5N1), and niches are customarily defined at relatively coarse spatial scales to avoid complexities of biotic interactions. The variables used to define it are described in Methods. Here, we use ecological niche modeling to provide a landscape-scale perspective on the ecological context of HPAI-H5N1 occurrences across the Middle East and northeastern Africa (Figure 1), following protocols developed in an earlier study in West Africa [10]. In the previous study, we associated HPAI-H5N1 case occurrences with month-to-month variation in 'greenness,' in the form of Normalized Difference Vegetation Index (NDVI) values derived from the Advanced Very High Resolution Radiometer (AVHRR) satellite, in an evolutionary-computing environment. We thus produced ecological niche models of HPAI-H5N1 occurrence that had significant predictive ability, suggesting that HPAI-H5N1 occurs under consistent and predictable environmental circumstances in West Africa. In this study, we demonstrate consistent, predictable environmental conditions associated with HPAI-H5N1 occurrences across the Middle East and northeastern Africa, albeit not without notable exceptions.

## Results

Most of the 9 tests conducted as part of this study indicated that independent test points coincided with ENM predictions significantly better than random expectations (see Additional file 2), although not without exceptions. In other words, in general, models based on known HPAI-H5N1 occurrences were able to anticipate spatial distributions of independent samples of HPAI-H5N1 based on their environmental attributes. The details of these test results follow.

### Predictivity across study region

**Figure 2:**
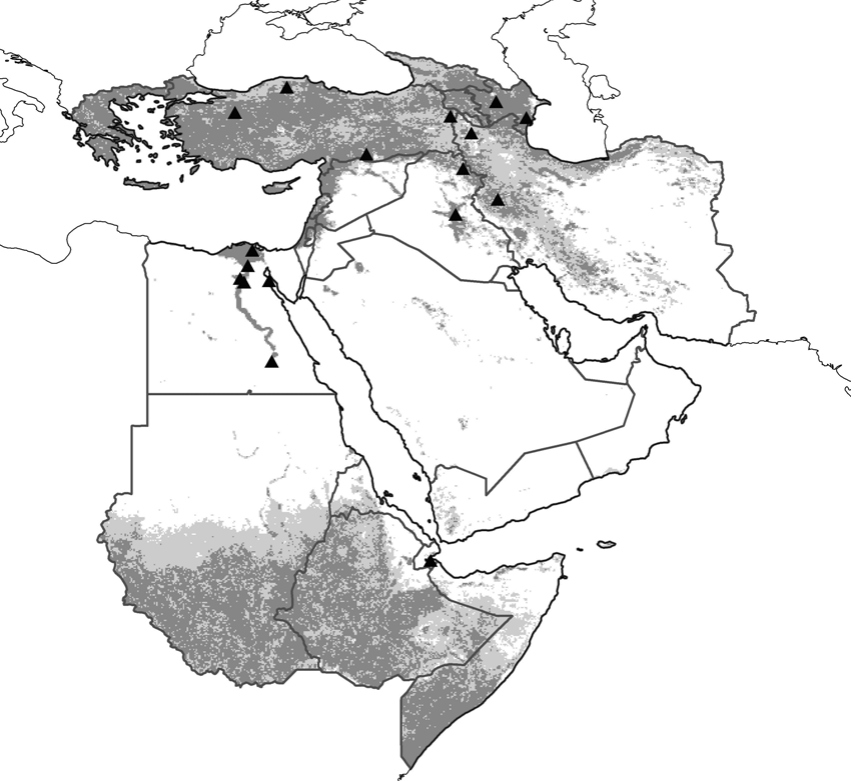
**Regional projection across the Middle East and northeastern Africa of HPAI-H5N1 ecological niche model results based on all OIE case occurrence points**. Model predictions are shown as ramps of model agreement in predictions: light grey = 5–9 models predict potential presence, dark grey = all models agree in predicting potential presence. Black triangles indicate independent test data (*N *= 17) from the region drawn from the ProMed archive of human case reports.

The model based on all OIE points showed significant predictive ability when tested with the ProMed human case-occurrence data (see Additional file 2; Figure 2). Potential for HPAI-H5N1 occurrence was predicted along the major rivers of the region (Euphrates, Nile, Tigris), across most of the Caucasus, southern Sudan, and in Ethiopia, Greece, northern and western Iran, southern Somalia, and Turkey. The virus was not predicted to have high probability of occurrence in the Sahara, nor more generally in arid areas. Egypt was largely predicted unsuitable, except for the fertile, densely populated Nile Valley. This model's predictions were significantly better than random expectations at all 10 thresholds; for example, at the 5 models out of 10 threshold, this model predicted 82.4% of the independent testing points in just 41.2% of the region (*P *< 0.001).

### Single testing regions

**Figure 3:**
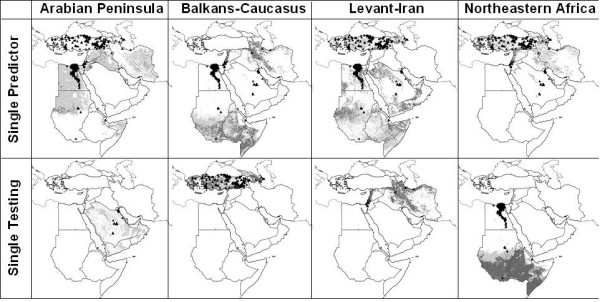
**Spatially stratified tests of ENM predictions of HP-H5N1 distributions in the Middle East and northeastern Africa**. Here, occurrences from each subregion predict distributions of cases in the rest of the region, and vice versa. Model predictions are shown as ramps of model agreement in predictions: light grey = 5–9 models predict potential presence, dark grey = all models agree in predicting potential presence. Only independent test points are plotted on maps. The dense cluster of testing points along the lower Nile River in northeastern Africa as testing region analyses covers an area predicted to be suitable.

These analyses tested the ability of models based on known occurrences across three subregions to predict patterns of occurrence in the fourth subregion. These tests indicated, for the most part, significant predictive power of the models (see Additional file 2; Figure 3). All thresholds of prediction were significant for prediction of occurrences in Levant-Iran by the remaining three regions, 8 of 10 thresholds were significant for predictions in northeastern Africa, and 7 of 10 were significant for predictions in Balkans-Caucasus. The model predicting distributions in the Arabian Peninsula performed more weakly than the other models, with only 4 of 10 thresholds significant and considerable deviation from coincidence when inspected visually (Figure 3).

### Single predictor regions

Predictions of independent points across landscapes based on single training regions were less successful (see Additional file 2; Figure 3). Indeed, only 2 of 4 models showed any predictive ability. Predictions from northeastern Africa to the rest of the region were significant at 8 of 10 thresholds, and projections from Levant-Iran to the rest of the region were significant at 5 of 10 thresholds. Projections based on models trained in the Arabian Peninsula and Balkans-Caucasus showed no significant ability when challenged to predict occurrences in the remaining regions. Once again, visually, the Arabian Peninsula models performed particularly poorly (Figure 3).

### Partial ROC analyses

The partial ROC analyses (see Additional file 2) were largely consistent with the cumulative binomial probability results (see Additional file 2). According to these tests, all single-testing-region predictions were successful (i.e., P ≤ 0.001) while 2 of 4 single predictor regions (Levant-Iran, northeastern Africa) were significantly better than random (P ≤ 0.005). The partial ROC evaluation of the overall prediction of the ProMed data was similarly significant (P < 0.01)

**Figure 4:**
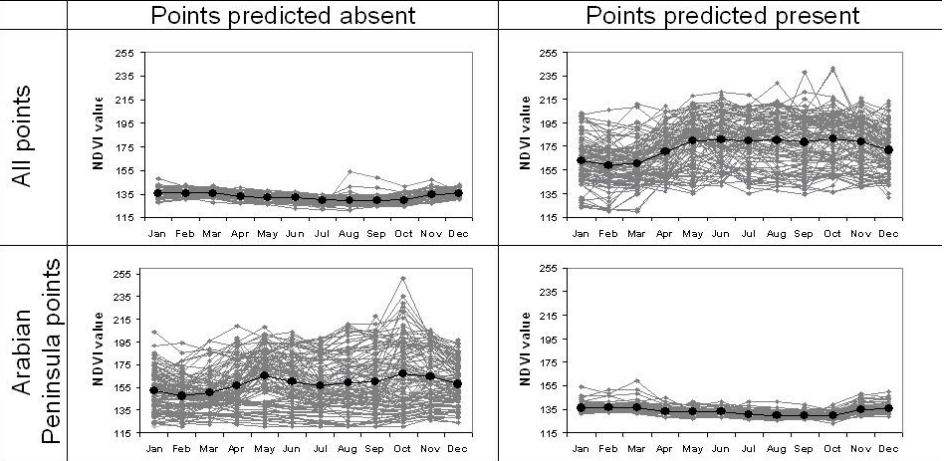
**Summary of Normalized Difference Vegetation Index (NDVI) 'greenness' profiles of the Middle East and northeastern Africa through one year for models based on the entire region (top) and for models based only on the Arabian Peninsula**. In each case, we show NDVI values for 100 randomly selected points of predicted absence versus 100 randomly selected points of predicted presence. Median values are shown in bold.

The NDVI data used in this study summarize photosynthetic mass of vegetation, and how this quantity changes through the year. Models based on case occurrences from across the region were compared in detail in terms of environmental conditions reconstructed as suitable versus unsuitable (Figure 4), approximating a visualization of the ecological niche estimated by each model. In the all-region model, HPAI-H5N1 was predicted absent from areas with low NDVI values and low seasonality, but present in areas with a broad range of NDVI values (from low to high) that showed marked seasonal variation. In contrast, the Arabian Peninsula model predicted presence in low NDVI areas with minimal seasonality, and absence from areas showing a broad range of NDVI values (from low to high) and seasonal variation. As such, the model with the least predictive ability (i.e., the Arabian Peninsula model) was the inverse of the one that had good predictive ability (i.e., the all-region model). It is interesting to compare these results to those from our previous West African models [10]. There, virus presence was predicted mostly in savannah and woodland habitats, whereas absence was predicted in montane areas, coastal mangroves, the freshwater swamps of the Niger Delta, and from rainforest areas: areas of highest predicted HPAI-H5N1 risk were highly variable seasonally, just as with our all-region model.

**Figure 5:**
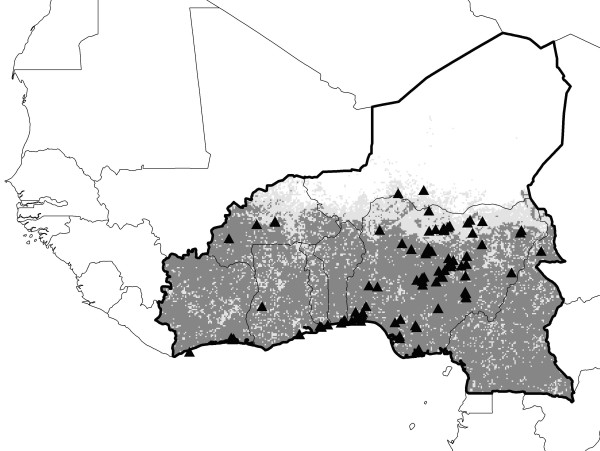
**Regional projection across West Africa of HPAI-H5N1 ecological niche model**. Results based on OIE case occurrence points and environmental layers for the Middle East and northeastern Africa. Model predictions are shown as ramps of model agreement in predictions: light grey = 5–9 models predict potential presence, dark grey = all models agree in predicting potential presence. Black diamonds indicate independent test data (*N *= 101) from the region [10,11]. Study area is delineated by bold border.

The spatial limits of the predictivity we have documented remain an open question [10]. The initial demonstration of predictable HPAI-H5N1 geography across West Africa is now supported by replication of the modeling protocol across the Middle East. Projection of the Middle East model to West Africa, and testing with independent points from that region [10,11] (*N *= 101;) demonstrated significant predictivity at all thresholds with both the binomial test, and the partial ROC approach. This new prediction (Figure 5) is broadly quite similar to the first West African prediction [10], although differences are evident. In particular, the Middle East model predicts HPAI-H5N1 presence in forest and mountains, whereas the West African model did not. The two models are based upon different sets of environmental layers, so some level of difference is not surprising.

## Discussion

Our results are generally consistent with earlier predictions of the ecological niche of HPAI-H5N1 in West Africa [10]. Most Middle Eastern and northeastern African models predicted suitable areas for HPAI-H5N1 transmission in human-habitable areas, such as the Nile Valley, the Levant, the Fertile Crescent, and the savannas of southern Sudan. The major difference between the two sets of models is that most Middle Eastern and northeastern African models predicted suitability in montane areas (Caucasus, Ethiopian Highlands, northern and western Iran, and Turkey), whereas the West African models focused prediction of suitable areas in lowlands. These models agree most clearly in implicating areas with greatest seasonal variation as representing high HPAI-H5N1 risk.

**Figure 6:**
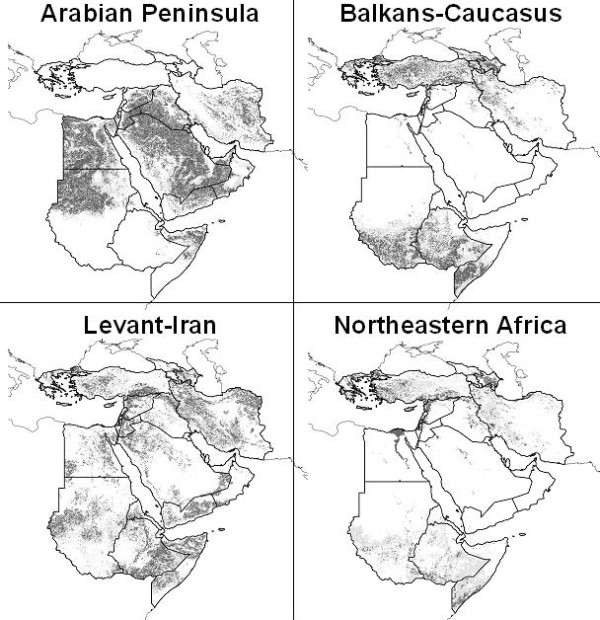
**Projections of HPAI H5N1 occurrences from a single subregion across the whole region**. Light grey = 5–9 models predict potential presence, dark grey = all models agree in predicting presence. Note the contrast between the Arabian Peninsula and the other three predictions.

The major exception to the conclusion of predictivity of HPAI-H5N1 in the Middle East and northeastern Africa were predictions involving the Arabian Peninsula, which were not generally statistically significantly better than random expectations. Indeed, in several areas, Arabian models were *inverse *to the rest of our predictions, predicting absence in areas of presence and vice versa. That is to say, models based on Arabian Peninsula points predicted HPAI-H5N1 presence in deserts, but not in mountains, the Levant, the Fertile Crescent, or in the Sudanese savannah, and only at low levels of model agreement in the Nile Valley (see Additional file 2; Figure 6).

It is interesting that Arabian models should produce predictions so inconsistent with those from the rest of the study area (see Additional file 2): for example, the distribution of Arabian Peninsula occurrences could not be predicted with any confidence by models trained in the remainder of the region, and conversely, Arabian Peninsula points were unable to predict occurrences across the Balkans, Caucasus, Levant, Iran, or northeastern Africa successfully. Three major HPAI-H5N1 outbreaks occurred in the Arabian Peninsula: in Kuwait, Ar-Riyad (city), and southern Ar-Riyad (province), none of which is predicted strongly by models trained elsewhere (Figures 2 and 3). Given the rather extreme arid conditions in the region, the Arabian Peninsula seems a harsh environment for both poultry and poultry diseases. We suspect that Arabian HPAI-H5N1 occurs chiefly or only in human-subsidized habitats that would permit poultry to be raised: indeed, 26 of 30 reported Saudi Arabian cases were detected in commercial farms containing thousands to hundreds of thousands of poultry [11]. Perhaps, Arabian occurrence points reflect something other than the "ecological niche" of HPAI-H5N1 in the subregion; for example, they may reflect principally the conditions under which poultry can be raised, albeit with considerable subsidy of water and shade, irrespective of disease distributions. We should add, though we suspect that such is not the case, the total lack of predictivity in the Arabian Peninsula raises the more troubling possibility that the correspondence between NDVI and disease occurrence in the rest of the region is coincidental. It is possible that HPAI H5N1 distribution is not driven by factors correlated with NDVI seasonality, but by something that cannot be detected in the remotely sensed landscape.

Gilbert et al. [12] mapped the geographic distribution of suitable conditions for HPAI-H5N1 across Southeast Asia, finding close associations between free-grazing domestic ducks in rice paddies and HPAI-H5N1 cases. This result suggests that transmission risk could be mapped successfully in Southeast Asia, where duck production and rice cultivation are both extensive and intertwined, and that duck production may be an important driver of HPAI-H5N1 persistence. The authors stated that large numbers of Anatidae concentrate in the Nile Delta, and that the Hadejia Jama'are river system of Nigeria is also an important area of duck production. FAO reports a combined domestic duck and goose population of 18.3 million for Egypt in 2004 [13], presumably concentrated in the Nile Delta and Valley (along with virtually the entire human population and all productive agricultural land), joined in winter by large flocks (several hundreds of thousands [14]) of wild aquatic birds. Figures are unavailable for domestic Anatidae in Nigeria, although numbers of undifferentiated "exotic poultry" (ducks, geese, turkeys, guinea-fowl, ostriches, etc.) in the 5 states bordering Hadejia Jama'are were around 7.5 million birds in 2003 [15]. Egypt and Nigeria both produce substantial rice crops (on 613 000 and 2 725 000 ha of land, respectively) [16].

Although total area under rice cultivation and total Anatidae populations are far higher in East Asia than in Egypt, the ratio of domestic Anatidae to area of rice production is considerably higher than in Thailand and Vietnam (see Additional file 3), and about the same as that found in China. If grazing of domestic Anatidae in rice paddies does play an important role in driving HPAI-H5N1 persistence and if duck-raising in the Middle East parallels that in East Asia, we might, expect persistence in China, Egypt, and Iran, all countries with higher duck-to-rice production area ratios than Thailand (Additional file 3). On the other hand, cases of HPAI-H5N1 have been numerous and widespread in Turkey, despite low numbers of Anatidae and little rice cultivation, suggesting that duck grazing in rice paddies is not the only factor in HPAI-H5N1 transmission and persistence. Free mingling of backyard poultry and wild birds has been identified as a risk factor for HPAI-H5N1 transmission [17,18]. In Egypt, most domestic Anatidae are considered to be backyard (64% of ducks and "all" geese), whereas the majority of chickens (63%) are produced in commercial operations, apparently typifying the poultry industry of North Africa and the Middle East [19].

**Figure 7:**
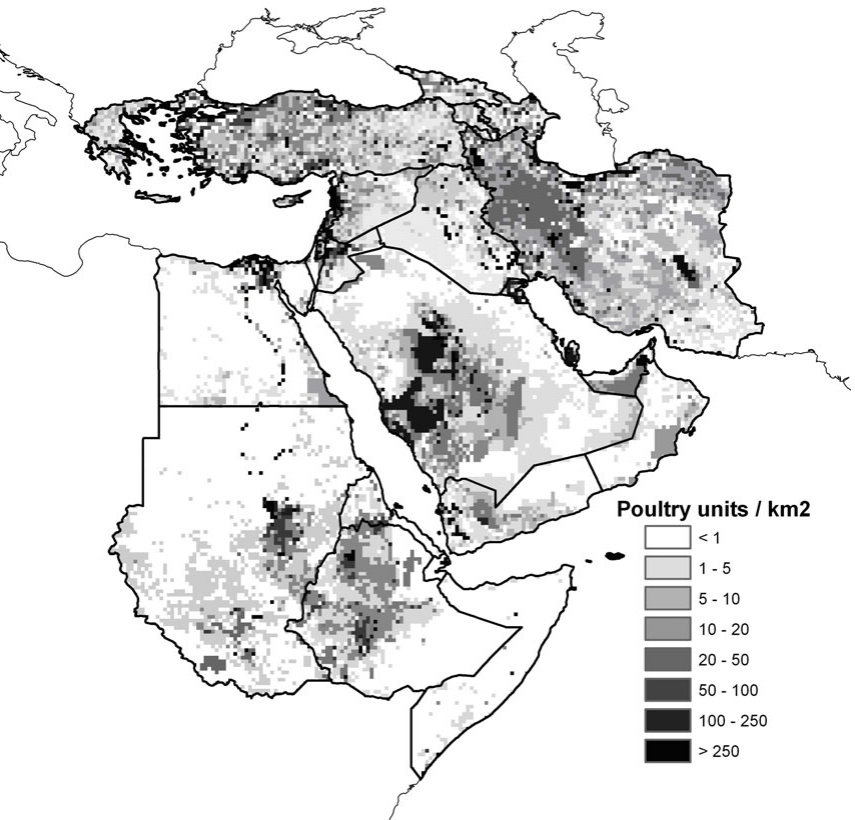
**Density of poultry in the Middle East and northeastern Africa (units per km^2^) **[13].

Our models and predictions cannot shed new light on the comparative roles of poultry and wild birds in HPAI-H5N1 transmission. One of the most important challenges for our analyses is distinguishing true ecological biases in case distributions (i.e., the ecological niche!) from the spatial and ecological biases in distributions of HPAI-H5N1 hosts. In some regions (Nile Delta, Fertile Crescent, Levant, Turkey, western Iran), our predictions showed marked coincidence with poultry distributions (Figure 7). However, our models failed to predict the high poultry concentrations in western Saudi Arabia and the Arabian Gulf states as forming part of the potential distribution of HPAI-H5N1, despite detections in Kuwait; as noted previously, our ability to predict HPAI-H5N1 distribution patterns in the Arabian Peninsula was poor in all comparisons.

## Conclusion

HPAI H5N1 detection data used for the development of these models are dominated by transmission among flocks of several poultry species. Given that detection data are so variable in terms of species composition (i.e., taxa, and number of taxa affected), husbandry method (high biosecurity, backyard, etc), origin (home-hatched, purchased, native-hatched, imported legally or illegally), and domestication, it is hard to define mechanisms driving transmission. We do not, however, find that our models are simply reproducing the spatial distributions of poultry flocks. Several ecologically-biased elements in the HPAI-H5N1 transmission cycle could explain the predictivity we detected: introduction of HPAI-H5N1 by migratory birds [20,21], transmission among poultry flocks [22,23], areas important for importation of poultry or hobby birds (legal or illegal) [24], or even transportation routes (e.g., roads, rivers). Inconsistencies in predictions based on HPAI-H5N1 occurrences from different subregions suggest that certain of these factors may have greater importance in some subregions than in others. In the Middle East, at least, we observe coincidence between human populations and HPAI-H5N1 cases, although, of course, this observation may simply point to the fact that influenza surveillance is more intensive in populated areas.

## Methods

### Ecological Niche Models

The ecological niche models (ENMs) developed in this study are based on the idea that organisms and other biological phenomena (including disease transmission) have distinct ecological requirements that determine their occurrences in time and space [25]. In general, disease applications of ENM balance between focusing on individual species in the transmission system and using the integration of the whole system as a "black box" determining transmission to some species or biological phenomenon of interest [26,27]. In this contribution, given the as-yet poorly characterized avian reservoir of HPAI-H5N1, we focus on all cases of HPAI-H5N1, effectively treating the transmission system as a black box. We thus attempt to model the transmission of a single pathogen based on its appearance in a multi-species system (i.e., the subset of animals in which HPAI H5N1 has been detected), in this case, dominated by distributions of domestic birds. In this sense, we deviate somewhat from the classical ENM approaches, which are based on single-species occurrence-environment correspondence. ENMs have been developed via diverse methodological approaches [28-31]; however, the method most frequently applied to questions of disease transmission has been the Genetic Algorithm for Rule-set Prediction (GARP), an evolutionary-computing approach [32,33].

### Input data

This study was based on HPAI-H5N1 animal case-occurrence data for 2005–2008 from the Middle East and northeastern Africa. Data were drawn from the World Organisation for Animal Health (OIE) [11], consisting of 610 unique locations, including isolations from wild birds, zoo birds, commercial poultry, and backyard poultry (Figure 1). This survey of occurrences includes birds assumed to be raised under strict biosecurity control, as well as birds raised with none; it similarly includes birds raised in strictly monospecific farms, multispecies assemblies mingling freely with wild birds (and other fauna), and even pets in a children's kindergarten. The database is composed of detections in at least 18 species of birds, although reporting standards are not consistent, so all too frequently information about hosts is either vague or absent. Most detections occurred in anthropogenic habitats. Our study area included 25 countries and one territory, ranging from Greece to the northwest, Somalia to the southwest, Georgia to the north, and Iran to the east. We assembled a complementary set of 17 unique and non-overlapping human cases occurrences from the archives of the International Society for Infectious Disease (ProMed Avian Influenza archive) [34] from the region (Figure 2) with which to test model predictions. All textual descriptions of occurrence localities were converted to geographic coordinates accurate to the nearest 0.01° using the GeoNet Names Server , Alexandria Digital Library Gazetteer , and other sources [35].

We based ENM development on the 610 OIE localities for which geographic coordinates were provided with a precision of at least 0.01°; duplicate localities (i.e., multiple occurrences in the same 8 × 8 km grid square) were discarded. Geographic coordinates in the OIE data set were drawn from global positioning system recordings for the point of detection of HPAI-H5N1 cases [11]. They thus specify the spatial position of HPAI-H5N1 occurrences, and probably represent the coarse-scale ecological conditions under which HPAI-H5N1 transmission occurs.

Given that the spatial pattern of H5N1 outbreaks has been on rather fine spatial scales, our previous experience with niche modeling and H5N1 outbreaks indicates that spatial resolutions on the order of 1–10 km are necessary, making use of climate-based data layers impractical. Environmental data sets included 12 monthly composite remotely-sensed data layers for Nov 1999 – Oct 2000, each summarizing maximum Normalized Difference Vegetation Index (NDVI; native spatial resolution 8 × 8 km) values [36]; although not exactly coincident with occurrence data temporally, these data provided an exemplar year of landscape variation in greenness. As NDVI is derived from reflectance in the visible and near-infrared domains, and as such is sensitive to photosynthetic activity and is closely correlated with photosynthetic mass [36], the NDVI time series used here summarizes aspects of land cover and vegetation phenology across the region. A year 2001 MODIS-based vegetation continuous fields dataset summarizing percent tree cover was also used (native spatial resolution 500 m) [37]. Finally, we also included 3 data sets summarizing aspects of topography: slope, aspect, and compound topographic index (which summarizes tendency to pool water), from the U.S. Geological Survey's Hydro-1K data set (native resolution 1 km) [38]. We deliberately excluded data on elevation from the study to avoid confusion caused by indirect variables. Climate data were not included in these analyses for lack of sufficiently high-resolution data sets across the region.

### The GARP algorithm

The Genetic Algorithm for Rule-set Prediction (GARP) has been applied widely to questions of disease transmission [26,39], and its predictive ability has been tested under diverse circumstances [30,40,41]. Although GARP has seen criticism in some comparative studies [30], more recent studies have indicated considerably better performance [42,43] and some artifactual causation of previous results [44]. As such, we used GARP for ENM development.

In general, we developed tests based on spatially stratified subsets of available occurrence information set aside prior to model development. Of occurrence data actually input into GARP, the program divides occurrence data randomly into three subsets: training data (25%; for rule development), intrinsic testing data (25%; for evaluation of rules) and extrinsic testing data (50%; for evaluation of model quality, see below). Spatial predictions of presence versus absence can include two types of error: false negatives (areas of actual presence predicted absent) and false positives (areas of actual absence predicted present) [45] – rule performance in each of these dimensions is evaluated via the intrinsic testing data set. Changes in predictive accuracy from one iteration to the next are used to evaluate whether particular rules should be incorporated into the model or not, and the algorithm runs either 1000 iterations or until convergence [33]. The final rule-set is then used to query the environmental data sets across the study region to identify areas fitting the rule set predictions to produce a hypothesis of the potential geographic distribution of the species [25].

Since GARP processing includes several random-walk components, each replicate model produces distinct results, representing alternative solutions to the optimization challenge. Following best-practices approaches [40], we developed 100 replicates of each model. We filtered these replicates based on their error characteristics, retaining the 20 with lowest false negative rates (= percentage of independent testing points falling in areas not predicted to be suitable), and then retained the 10 (of the 20) closest to the median of proportional area predicted present, an index of false-positive error rates [40]. A consensus of these 'best subset' models was then developed by summing values for each pixel in the map to produce final predictions of potential distributions with 11 thresholds (integers from 0 to 10).

### Modeling and testing approach

This study focuses on the question of whether HPAI-H5N1 transmission in the Middle East and northeastern Africa occurs under a consistent and predictable set of environmental conditions. As such, we developed a series of tests of model predictivity; in each case, models were developed and predictions tested using spatially independent suites of occurrence data. Model tests were based on 4 spatial subsets of the Middle Eastern and northeastern African occurrence data (Figure 1): Arabian Peninsula (Bahrain, Kuwait, Oman, Qatar, Saudi Arabia, United Arab Emirates, Yemen; *N *= 31), Balkans-Caucasus (Armenia, Azerbaijan, Cyprus, Georgia, Greece, Turkey; *N *= 175), Levant-Iran (Iran, Iraq, Israel, Lebanon, Palestinian Territories, Syria; *N *= 18), and northeastern Africa (Djibouti, Egypt, Eritrea, Ethiopia, Somalia, Sudan; *N *= 386).

The basic design of testing included three schemes for subdividing available occurrence data, as follows:

1. *Single testing regions*: We combined each possible set of 3 subregional occurrence datasets to develop ENMs that were tested with the fourth subregion. Total 4 tests.

2. *Single predictor regions*: Occurrence data for each subregion were used to develop predictive models that were projected to the rest of the region for testing (e.g., Arabian Peninsula data points used to build predictions for the combination of Levant-Iran, northeastern Africa, and Balkans-Caucasus). Total 4 tests.

3. *Predictivity across study region*: We developed ENM predictions based on all OIE veterinary cases in the region, and tested its prediction based on coincidence of predictions with the 17 independent ProMed human cases. One test.

The customary approaches to spatial model validation (e.g. simple receiver operating characteristic, kappa statistics) are not applicable to situations in which presence-only data are the only information available [45,46]. As such, we validated models using two approaches. First, we calculated binomial probabilities that observed coincidence of predictions and independent test data is no better than random, with the probability of *k *successes in *N *trials depending on *p*, the probability of success in any one trial; we estimated *p *as the proportion of the testing area predicted present, and *k *as the number of the *N *testing points successfully predicted by the model prediction [40]. Binomial probabilities were calculated for each of the 10 thresholds representing predictions of presence (1 = broad, 10 = narrow), in each case testing whether predictivity was better than expected by chance.

Second, we followed Phillips et al. [47] in modifying receiver operating characteristic curves (ROCs) so as not to depend on absence data. We calculated the area under the curve (AUC) of the ROC, a statistical technique that has become a dominant tool in evaluating the accuracy of models predicting distributions of species ^16^. However, when comparing two ROCs, AUC systematically undervalues models that do not provide predictions across the entire spectrum of proportional areas in the study area (such as GARP, the modeling approach used here) [48]. In addition current ROC approaches inappropriately weight the two error components (omission and commission) equally. Accordingly, we use a modification of ROC that remedies these problems: partial-area ROC approaches that evaluate only over the spectrum of the prediction and that allow for differential weighting of the two error components [48].

We carried out partial ROC analyses [48] for each model, all based solely on independent testing points not used to train the models in areas from distinct regions(s) to which models were projected. AUCs were limited to the proportional areas over which models actually made predictions, and only omission errors of <5% were considered (i.e., E = 5% [48]). We calculated partial AUCs using a program based on the trapezoid method [49] kindly developed by N. Barve (in prep.), and present our ROC results as the ratio of the model AUC to the null expectation ("AUC ratio") [48]. Bootstrapping manipulations to permit evaluation of statistical significance of AUCs (as compared with null expectations) were achieved by resampling 50% of the test points with replacement 1000 times from the overall pool of testing data; one-tailed significance of differences in AUC (i.e. elevation above the line of null expectation) was assessed by counting the number of bootstrap replicates with AUC ratios <1.

## Competing interests

The authors declare that they have no competing interests.

## Authors' contributions

Conceived and designed the experiments: ATP, RW. Analyzed the data: RW. Drafted the manuscript: ATP, RW. All authors read and approved the final manuscript. ATP and RW are guarantors of the paper.

## Supplementary Material

Additional file 1 **Summary of HPAI-H5N1 detections from countries across the Middle East and northeastern Africa reported by OIE^a ^**[4], **ProMed^b ^**[34], **and WHO^c^**[5]. Note that numbers of wild bird cases seem to be unreliable: on one hand, these numbers are overreported in Egypt, where cases in birds captive in Giza Zoo are counted as "wild", and probably underreported from Azerbaijan, where "die-offs" yielded only 3 positive detections. Clearly, however, poultry cases far outnumber wild cases, and numbers of birds culled to prevent disease spread are higher still. Click here for file

Additional file 2 **Summary of model predictions, binomial tests and partial ROC tests in this study, illustrated by information for the threshold >5 of 10 best subsets models predicting potential for presence**. "Prop. area" indicates the proportion of the test region predicted present at that threshold. Also provided is the number of thresholds (out of 10) for which model predictions were significantly better than random expectations. Values under Max, Min, and Mean characterize distributions of AUC ratios (maximum, minimum, and mean) across 1000 bootstrap replicates, and the number of bootstrap replicates falling at or below unity. Click here for file

Additional file 3 **Comparison of populations of domestic Anatidae and area under rice cultivation in 5 HPAI-H5N1 affected countries**. Data drawn from ^a^Food and Agriculture Organization – Global Livestock Production and Health Atlas [13] and ^b^International Rice Research Institute [16]. Click here for file
