# Supplementary material for: Ecology and geography of avian influenza (HPAI H5N1) transmission in the Middle East and northeastern Africa
Source: Int J Health Geogr. 2009 Jul 20;8:47. doi: 10.1186/1476-072X-8-47 (PMC2720944; doi:10.1186/1476-072X-8-47)
Supplement: Additional file 1 — Summary of HPAI-H5N1 detections from countries across the Middle East and northeastern Africa reported by OIEa [4], ProMedb [34], and WHOc[5]. Note that numbers of wild bird cases seem to be unreliable: on one hand, these numbers are overreported in Egypt, where cases in birds captive in Giza Zoo are counted as "wild", and probably underreported from Azerbaijan, where "die-offs" yielded only 3 positive detections. Clearly, however, poultry cases far outnumber wild cases, and numbers of birds culled to prevent disease spread are higher still. [file 1476-072X-8-47-S1.doc]

**Additional file 1.**

**Table 1.**

| Country | Date of first detection ^a^ | Species of first detection ^b^ | No. of human cases ^c^ | No. of wild cases ^a^ | No. of poultry or captive bird cases ^a^ | No. of poultry culled ^a^ |
| --- | --- | --- | --- | --- | --- | --- |
| Turkey | 6 Oct 2005 | *Meleagris gallopavo* | 12 | 16 | 9178 | 289 812 |
| Kuwait | 11 Nov 2005 | *Phoenicopterus ruber* | 0 | 0 | 131 | 466 996 |
| Iraq | 9 Jan 2006 | *Homo sapiens* | 3 | 0 | 652 | 3478 |
| Azerbaijan | 29 Jan 2006 | *Cygnus* sp. | 8 | 3 | 1 | 296 000 |
| Iran | 2 Feb 2006 | *Cygnus* sp. | 0 | 153 | 14 | 475 |
| Greece | 9 Feb 2006 | *Cygnus* sp. | 0 | 17 | 0 | 0 |
| Egypt | 17 Feb 2006 | Poultry | 50 | 19 | 1 075 920 | 8 840 215 |
| Sudan | 20 Feb 2006 | Poultry | 0 | 0 | 87 370 | 107 327 |
| Georgia | 23 Feb 2006 | *Cygnus* sp. | 0 | 10 | 0 | 0 |
| Israel | 16 Mar 2006 | *Gallus gallus*, *Meleagris gallopavo* | 0 | 0 | 15 212 | 256 414 |
| Palestinian Terr. | 23 Mar 2006 | Poultry | 0 | 0 | 5000 | 40 800 |
| Jordan | 23 Mar 2006 | *Gallus gallus*, *Meleagris gallopavo* | 0 | 0 | 21 | 18 000 |
| Djibouti | 5 Apr 2006 | Local poultry | 1 | 0 | 4 | 18 |
| Saudi Arabia | 12 Mar 2007 | *Struthio camelus* | 0 | 0 | 12 606 | 5 310 290 |
| **TOTALS** |  |  | 74 | 218 | 1 206 109 | 15 629 825 |
