# Supplementary material for: Ecology and geography of avian influenza (HPAI H5N1) transmission in the Middle East and northeastern Africa
Source: Int J Health Geogr. 2009 Jul 20;8:47. doi: 10.1186/1476-072X-8-47 (PMC2720944; doi:10.1186/1476-072X-8-47)
Supplement: Additional file 2 — Summary of model predictions, binomial tests and partial ROC tests in this study, illustrated by information for the threshold >5 of 10 best subsets models predicting potential for presence. "Prop. area" indicates the proportion of the test region predicted present at that threshold. Also provided is the number of thresholds (out of 10) for which model predictions were significantly better than random expectations. Values under Max, Min, and Mean characterize distributions of AUC ratios (maximum, minimum, and mean) across 1000 bootstrap replicates, and the number of bootstrap replicates falling at or below unity. [file 1476-072X-8-47-S2.doc]

**Additional file 2.**

|  | Sample size (train/test) | Prop. area | Number of successes | Cumulative binomial probability | Number of thresholds significant | Max | Min | Mean | Number of replicates ≤1 |
| --- | --- | --- | --- | --- | --- | --- | --- | --- | --- |
| **Single-testing region** |  |  |  |  |  |  |  |  |  |
|  |  |  |  |  |  |  |  |  |  |
| Arabian Peninsula / Levant-Iran / northeastern Africa → Balkans-Caucasus | 435/176 | 0.836 | 158/175 | 0.004 | 7 | 1.611 | 1.140 | 1.457 | 0 |
| Arabian Peninsula / Balkans-Caucasus / northeastern Africa → Levant-Iran | 592/18 | 0.412 | 13/18 | 0.002 | 10 | 1.708 | 1.071 | 1.217 | 0 |
| Arabian Peninsula / Balkans-Caucasus / Levant-Iran → northeastern Africa | 224/386 | 0.524 | 277/386 | 0.000 | 8 | 1.081 | 1.012 | 1.035 | 0 |
| Balkans-Caucasus / Levant-Iran / northeastern Africa → Arabian Peninsula | 579/31 | 0.355 | 12/31 | 0.282 | 4 | 1.271 | 1.021 | 1.065 | 0 |
| **Single predictor region** |  |  |  |  |  |  |  |  |  |
| Arabian Peninsula → Balkans-Caucasus / Levant-Iran / northeastern Africa | 31/579 | 0.351 | 69/579 | 0.999 | 0 | 1.002 | 0.991 | 0.995 | 997 |
| Balkans-Caucasus → Arabian Peninsula / Levant-Iran / northeastern Africa | 186/435 | 0.260 | 101/435 | 0.902 | 0 | 0.981 | 0.976 | 0.977 | 1000 |
| Levant-Iran → Arabian Peninsula / Balkans-Caucasus / northeastern Africa | 18/592 | 0.428 | 404/592 | 0.000 | 5 | 1.656 | 0.951 | 1.272 | 2 |
| Northeastern Africa → Arabian Peninsula / Balkans-Caucasus / Levant-Iran | 386/224 | 0.230 | 124/224 | 0.000 | 10 | 1.120 | 0.996 | 1.043 | 1 |
| **Predictivity across study region** |  |  |  |  |  |  |  |  |  |
| OIE veterinary cases → ProMed human cases | 610/17 | 0.412 | 14/17 | 0.000 | 10 | 1.657 | 0.992 | 1.209 | 9 |
