# Supplementary material for: Ecology and geography of avian influenza (HPAI H5N1) transmission in the Middle East and northeastern Africa
Source: Int J Health Geogr. 2009 Jul 20;8:47. doi: 10.1186/1476-072X-8-47 (PMC2720944; doi:10.1186/1476-072X-8-47)
Supplement: Additional file 3 — Comparison of populations of domestic Anatidae and area under rice cultivation in 5 HPAI-H5N1 affected countries. Data drawn from aFood and Agriculture Organization – Global Livestock Production and Health Atlas [13] and bInternational Rice Research Institute [16]. [file 1476-072X-8-47-S3.doc]

**Additional file 3.**

|  | Total poultry, 2004 ^a^ | Domestic Anatidae, 2004^a^ | Area of rice cultivation, 2006 ha^b^ | Anatidae / rice ha |
| --- | --- | --- | --- | --- |
| China | 4 735 229 952 | 875 230 000 | 29 380 000 | 29.79 |
| Egypt | 112 150 000 | 18 300 000 | 613 000 | 29.85 |
| Iran | 284 600 000 | 2 600 000 | 620 000 | 4.19 |
| Thailand | 187 270 000 | 17 270 000 | 10 073 000 | 1.71 |
| Vietnam | 252 000 000 | 75 000 000 | 7 324 000 | 10.24 |
